# Patterns of health-risk behaviors among Chinese adolescents during the COVID-19 pandemic: a latent class analysis

**DOI:** 10.1186/s12889-025-22089-5

**Published:** 2025-03-25

**Authors:** Mingxiu Liu, Xiaolei Tang, Qingyun Xia, Xiaoman Wu, Yinmei Yang, Hong Xiang, Jun Hu

**Affiliations:** 1 School of Nursing, Hubei University of Medicine, Shiyan City, 442000 People’s Republic of China; 2 Department of Burns and Plastic Surgery, Taihe hospital, the affiliated hospital of Hubei University of Medicine, Shiyan City, 442000 People’s Republic of China; 3 College of Public Health, Zhengzhou University, 100 Kexue Road, Gaoxin district, Zhengzhou City, 450001 People’s Republic of China; 4 Henan Key Laboratory of Chronic Disease Prevention and Therapy & Intelligent Health Management, Zhengzhou City, 450000 People’s Republic of China

**Keywords:** Health-risk behaviors, Adolescents, Latent class analysis, Influencing factors

## Abstract

**Background:**

Adolescent health-risk behaviors are prevalent and tend to co-occur. This study aimed to identify patterns of health-risk behaviors among Chinese adolescents during the COVID-19 pandemic and explore the effects of individual and social factors on health-risk patterns.

**Methods:**

This cross-sectional study investigated 1607 adolescents from four high schools in 2021 through stratified cluster random sampling. Latent class analysis was conducted to identify patterns of health-risk behaviors and logistic regression was used to examine the risk and protective factors of latent class membership.

**Results:**

Four latent classes were identified: “Low risk” (81.6%), “Problematic Internet use” (7.8%), “Alcohol use” (8.5%), and “High risk” (2.1%). Relative to the “Low risk”, adolescents with higher levels of sensation seeking, deviant peer affiliation, and childhood abuse were more likely to be assigned to the “Problematic Internet use” class, while those with high degrees of parental monitoring and school connectedness were less likely to be in the “Problematic Internet use” class. Those with higher levels of sensation seeking and deviant peer affiliation, lower scores of parental monitoring and school connectedness were more likely to be assigned to the “Alcohol use” class, compared to the “Low risk”. Students in the “High risk” class were more likely to report higher levels of sensation seeking, deviant peer affiliation, and childhood abuse, but lower degrees of parental monitoring and school connectedness than the “Low risk” class.

**Conclusions:**

This study identified patterns of multiple risk behaviors among Chinese high school students during the COVID-19 pandemic and found that multi-level individual and social factors affected latent classes of adolescent health-risk behaviors. These findings provide clues for designing effective interventions to reduce health-risk behaviors among adolescents.

## Introduction

### Health-risk behaviors in adolescence

Adolescence is a critical developmental stage characterized by heightened vulnerability to engaging in health-risk behaviors including bullying, smoking, alcohol consumption, and early sexual activity [1, 2]. Health-risk behaviors are prevalent among Chinese adolescents. For example, a study with a large sample size (*n* = 27019) conducted in 2022, in Zhejiang Province, China documented that 3.9% and 16.0% of adolescents smoked cigarettes and consumed alcohol in the past 30 days, and 13.7% of adolescents reported they had been involved in a physical fight within the past 12 months [3]. A cross-sectional survey found that 29.9% of Chinese adolescents met the criteria for possible problematic Internet use [4]. Also, a multicenter survey revealed that 9.75%, 10.57%, 15.17% of Chinese adolescents engaged in skipping school, running away from home, and fighting, respectively [5]. Further, previous studies have revealed that health-risk behaviors can lead to elevated risks of suicidality [6], anxiety and depression [7].

The COVID-19 pandemic introduced unprecedented disruptions to the lives of adolescents, as in-person schooling was replaced with online learning. This significant shift may be considered a traumatic event, potentially amplifying health-related risk behaviors, such as tobacco use, alcohol consumption, and increased electronic screen time [8, 9]. Therefore, it is essential to investigate adolescents’ health-risk behaviors during the COVID-19 pandemic, and elucidating the key determinants is informative for developing prevention and intervention strategies.

### Patterns of adolescent health-risk behaviors

Previous studies typically combine health-risk behaviors into a composite score assuming equal weights or explore health-risk behaviors separately ignoring the co-occurrence and heterogeneity based on variable-centered approaches [10, 11]. Problem Behavior Theory developed by Jessor [12] noted that co-occurring risk behaviors were prevalent in adolescence. Latent class analysis (LCA), a person-centered method, has an advantage in exploring the clustering of risk behaviors [13]. To date, several studies have discovered distinctive subgroups of health-risk behaviors based on LCA. For instance, a survey investigated health-risk behaviors among Thai secondary school students and found three latent classes, including the low-risk (88%), moderate-risk (11%), and high-risk classes (0.6%) [14]. Based on UK Millennium Cohort Study (*n* = 17223), Picoito and colleagues also explored adolescent substance use and antisocial behavior (e.g., smoking, drinking, cannabis use, physical fighting, shoplifting, vandalism, and graffiti), and identified four latent classes, including the “normative” (71.8%), “alcohol and physical fighting” (15.3%), “alcohol and tobacco” (9.9%) and “Poly-substance use and antisocial behaviors” classes (3.0%) [15]. Although the aforementioned studies have identified patterns of health-risk behaviors among adolescents, they ignore other prevalent health-risk behaviors in modern society, such as problem Internet use. Thus, the current study offers a more comprehensive understanding of patterns of adolescents’ multiple risk behaviors in Chinese context during the COVID-19 pandemic.

### Impacts of multi-level factors on adolescent health-risk behaviors

According to the Ecological Theory [16], individual factors and multiple social contexts play crucial roles in individuals’ health. At the individual level, sensation seeking refers to a personality trait characterized by a willingness to take risks in response to challenges, such as novel and complex experiences and strong emotions [17]. According to the Sensation Seeking Theory [18], high-sensation-seeking individuals tend to enjoy novel experiences and taking risks. A prior study found that urban and male adolescents as well as adolescents in non-intact families reported more health-risk behaviors [19]. In addition, a systematic review revealed that low parental education was a risk factor of drug abuse among adolescents [20]. Also, the prevalence of adolescent health-risk behaviors differed by age and grade level in school [21]. Therefore, guided by previous research, some socio-demographic variables concerning sex, grade, living places, age, maternal education, paternal education and family structure were adjusted in the current study.

Further, adolescents’ behaviors are embedded within social contexts, such as families, peers, and schools [22]. At the family level, childhood abuse is a crucial contributor to the prevalence of health-risk behaviors in adolescents [23]. Household dysfunction, a significant indicator of adverse childhood experiences, includes household mental illness, household substance abuse, household poverty, etc [24]. For example, adolescents exposed to parental mental illness and/or substance use disorder confront an elevated vulnerability to report substance use disorder [25]. Parental monitoring refers to parental awareness, watchfulness, and supervision of adolescent activities in multiple domains (i.e., friends, school, and behaviors at home), and communication to the adolescent that the parent is concerned about, and aware of those activities [26]. Parental monitoring can reduce adolescent sexual behaviors, substance use, and violence [27].

Adolescence as the stage when a second process of separation individuation takes place, during which they tend to individuate away from their family while becoming more susceptible to peer influence [28]. Adolescents prefer selecting peers who are similar to themselves in important ways, due to homophily selection [29]. Both cross-sectional and longitudinal studies have identified deviant peer affiliation as a robust predictor of adolescent problem behaviors [30, 31]. At the school level, the connection to school environment is a particularly protective factor against health-risk behaviors [32]. Prior research explored two dimensions of school connectedness and found that teacher support protected against the initiation of health-risk behaviors [33].

While some literature has demonstrated the association between individual, family, peer, school factors and adolescents’ involvement in risk behaviors, the distinct effects of multiple contexts on patterns of health-risk behaviors remain less well understood. Therefore, this study aims to address two research questions: (1) Are there distinct patterns or classes of adolescent health-risk behaviors? (2) How do individual, peer, family, and school factors simultaneously affect those latent classes?

## Methods

### Study design and participants

The following formula is used to calculate the sample size.

$$\:\text{n}={\text{Z}}_{1-{\upalpha\:}/2}^{2}\times\:\left[\text{P}\times\:\left(1-\text{P}\right)\right]/{\text{d}}^{2}$$

α = 0.05, $\:{\text{z}}_{1-{\upalpha\:}/2}$=1.96, $\:\text{d}=0.1\text{p}$, p is expected prevalence of health-risk behaviors among Chinese adolescents. Based on a survey conducted in eight provinces in 2021, 22.2% of Chinese adolescents engaged in high-risk behaviors [34]. The formulae yielded an initial sample size of 1346 students.

Four senior high schools were chosen to recruit participants in Shangqiu city in central China from May to June 2021. With the assistance of teachers, four or five classes were selected randomly from Grades 10 and 11 in each school. This study did not investigate adolescents from grade 12, as Chinese national college entrance exam is typically held on June 7 and 8. All students in the selected classes were invited to complete paper-based questionnaires. To gain rapport with participants, an orientation regarding data anonymity, objectives, and significance of the study was conducted. A total of 1650 questionnaires from 33 classes were collected. Participants with missing information on key variables (*n* = 43) were excluded, and 1607 respondents were eligible for final analysis.

### Measures

#### Health-risk behavior

Ten dichotomous questions (Yes or No) were used to measure whether adolescents engaged in following behaviors in the last 6 months, including skipping school, carrying weapons, engaging in fights, smoking cigarettes, drinking alcohol, cheating in tests, problematic Internet use, running away from home, vandalism, and sexual behavior, which were treated as observed indicator variables to identify latent classes. Response of “Yes” means that the student had engaged in the behavior one or more times in the in the last 6 months.

#### Sensation seeking

Sensation seeking was measured using the 8-item Brief Sensation Seeking Scale for Chinese (BSSS-C) (e.g., “I get restless if I do the same thing for a long time”) [35], which had adequate reliability and validity in Chinese adults (Cronbach’s α  = 0.90, Comparative Fit Index = 0.98, Standardized Root Mean of Residuals = 0.03). Each item was assessed on a 5-point Likert scale (1 = strongly disagree, 2 = disagree, 3 = neither agree nor disagree, 4 = agree, 5 = strongly agree). The higher composite score demonstrated higher sensation seeking. In the current study, the internal consistency was 0.71.

#### Deviant peer affiliation

15 items adapted from the National Youth Survey were used to estimate the affiliation with deviant peers in the last year [36], which has been validated in Chinese adolescents (Cronbach’s α was over 0.85) [37]. Sample items included “How many of your friends engaged in a fight in the last year?” “How many of your friends smoked cigarettes in the last year?” Responses were rated on a 3-point Likert scale (1 = none, 2 = some, 3 = most). A composite score was calculated, and higher scores suggested more deviant friends. In the current study, the internal consistency was 0.86.

#### Parental monitoring

The 8-item Parental Monitoring Scale was used to assess parental monitoring (e.g., “If I am going to be home late, I tell my parents/guardian”) [38], with a good internal consistency in a previous study (Cronbach’s α = 0.86) [1]. Responses ranged from 1 (Never) to 5 (Always). Higher composite scores represented more stringent parental monitoring. In the present study, the internal consistency was 0.85.

#### School connectedness

Based on previous tools [39, 40], 10 items were used to assess school connectedness, including teacher support (3 items), school belonging (3 items), and classmate support (4 items), which has been validated in Chinese high school students [41]. Sample items included “The teachers at this school treat students fairly?” Items were scored on a 5-point Likert scale ranging from “strongly agree” (1) to “strongly disagree” (5). Higher scores reflected greater school connectedness. In the current study, the internal consistency was 0.86.

#### Childhood abuse

Childhood abuse was assessed based on the Childhood Trauma Questionnaire-Short Form (CTQ-SF) [42], and it showed good reliability and validity in the Chinese adolescents (Comparative Fit Index = 0.91, Tucker-Lewis Index = 0.90, Root Mean Square Error of Approximation = 0.06, Cronbach’s α = 0.87) [43]. This study used 15 items to measure emotional abuse (e.g., “called stupid, lazy or ugly by family”), sexual abuse (e.g., “was molested”), and physical abuse (e.g., “was hit hard by family”). The response options ranged from 1 (never) to 5 (always). The Cronbach’s α was 0.78 in the current study.

#### Household dysfunction

Questions about household dysfunction came from Centers for Disease Control and Kaiser ACE Study [44]. Household dysfunction was evaluated by the endorsement of the following six experiences (Yes or No) during childhood: lived with anyone who had a problem with alcohol, drug or gambling, parental separation or divorce, witnessed domestic violence, mental illness in household, incarcerated household members, and family financial difficulties. Adolescents were defined as exposed to household dysfunction if they responded “Yes” to any item.

Other factors were also collected including sex (male or female), age (years), grade (10 or 11), living places (urban or rural), maternal and paternal education (elementary school or below, junior high school, senior high school, college or above), and family structure (intact family or non-intact family).

### Statistical analysis

First, the categorical variables were summarized as percentages, and continuous variables were expressed as means and standard deviations. Next, LCA with two through five latent classes were conducted using observed dichotomous variables (10 health-risk behaviors listed in Table 1). Model fit was assessed using a combination of Akaike information criterion (AIC), Bayesian information criterion (BIC), sample-size adjusted BIC (aBIC), Entropy, the Lo-Mendell-Rubin likelihood ratio (LMRLR), the adjusted Lo-Mendell-Rubin likelihood ratio (aLMRLR), the Bootstrap Likelihood Ratio Test (BLRT), class size, as well as interpretability [45]. Lower AIC, BIC, aBIC indicate superior fit, and small changes of AIC, BIC, aBIC with increased classes can also be considered as a criterion. Entropy ranged from 0 to 1, with values closer to 1 suggesting greater classification accuracy [46]. Entropy of 0.8 indicates approximately 90% correct group assignment [47], and entropy values above 0.8 are considered acceptable. There were no widely accepted standards for determining the number of samples in each class. Typically, researchers recommended that each class should comprise at least 50 cases and represent no less than 5% of the overall sample [48]. Nonetheless, some studies have incorporated classes with sizes below this 5% threshold or fewer than 50 cases [49]. When determining if a class size is too small, it’s crucial to examine whether the model fit statistics support the selected model and whether the small class is conceptually meaningful. Additionally, researchers must take into account the total sample size when evaluating the appropriateness of each class size [50]. Significant *p*-values of LMRLR, aLMRLR, and BLRT indicated that K classes was better than K-1 classes. After we identified the optimal model, students were assigned to each latent class based on the highest posterior probability. Finally, multinomial logistic regression was used to examine the risk and protective factors of latent class membership. LCA was carried out in Mplus 8.0. Other analyses were performed in SPSS 26.0. A two-sided *p* < 0.05 was considered statistically significant.

**Table 1:** Descriptive statistics (*n* = 1607)

Variables	*n*(%) or Mean ± SD
***Health risk behaviors***	
Skipping school	147(9.1)
Carrying weapons	54(3.4)
Engaging in fights	53(3.3)
Smoking cigarettes	93(5.8)
Drinking alcohol	248(15.4)
Cheating in tests	189(11.8)
Problematic Internet use	384(23.9)
Running away from home	54(3.4)
Vandalism	31(1.9)
Sexual behavior	38(2.4)
***Individual level***	
Male	853(53.1)
Grade	
10	745(46.4)
11	862(53.6)
*Rural	800(50.0)
Age (years)	16.3 ± 0.9
Sensation seeking	19.2 ± 4.3
***Peer level***	
Deviant peer affiliation	18.4 ± 3.6
***Family level***	
Maternal education	
Elementary school or below	399(24.8)
Junior high school	572(35.6)
Senior high school	356(22.2)
College or above	280(17.4)
Paternal education	
Elementary school or below	189(11.8)
Junior high school	663(41.3)
Senior high school	429(26.7)
College or above	326(20.3)
Intact family	1495(93.0)
Household dysfunction	446(27.8)
Parental monitoring	33.8 ± 5.4
Childhood abuse	17.5 ± 3.8
***School level***	
School connectedness	35.9 ± 6.9

Note: *8 adolescents did not report. SD, Standard Deviation

## Results

### Descriptive statistics

Table 1 describes the key study variables. The average age was 16.3 years, 53.1% of the sample were male, half of participants came from rural (50.0%), and 46.4% were in grade 10. The majority were from intact families (93.0%), and 27.8% of students experienced household dysfunction during childhood. Problematic Internet use (23.9%) was the most common health-risk behavior in the last 6 months, while vandalism (1.9%) and sexual behavior (2.4%) were the least prevalent.

### Patterns of adolescent health-risk behaviors

As shown in Table 2, the improvement in AIC was minimal after the 4-class model. The 3-class and 4-class models had the lowest BIC and aBIC, respectively. LMRLR and aLMRLR in the 5-class model with non-significant *p*-value, indicating that the 4-class model was more favorable than 5-class model. The entropy value of 4-class model was 0.819, indicating an acceptable level of class separation. While the smallest class consisted of 2.1% of adolescents in the 4-class model, the four identified classes represented empirically significant patterns of adolescent risk behaviors, each with plausible interpretations. Hence, based on a combination of model fit indices, the 4-class solution was selected as the optimal model.

**Table 2:** Model selection statistics of latent class analysis (*n* = 1607)

#Classes	AIC	BIC	aBIC	Entropy	LMRLR (*p*)	aLMRLR (*p*)	BLRT (*p*)	Smallest class
2	7338.738	7473.291	7393.870	0.900	< 0.001	< 0.001	< 0.001	11.0%
3	6971.027	7143.255	7041.597	0.845	< 0.001	< 0.001	< 0.001	2.1%
4	6937.626	7169.058	7032.455	0.819	0.033	0.035	< 0.001	2.1%
5	6929.832	7220.466	7048.919	0.865	0.173	0.177	0.030	1.6%

Note: AIC, Akaike information criterion; BIC, Bayesian information criterion; aBIC, sample-size adjusted BIC; LMRLR, the Lo–Mendell–Rubin likelihood ratio; aLMRLR, the adjusted Lo–Mendell–Rubin likelihood ratio; BLRT, Bootstrap likelihood ratio test

**Fig. 1:**
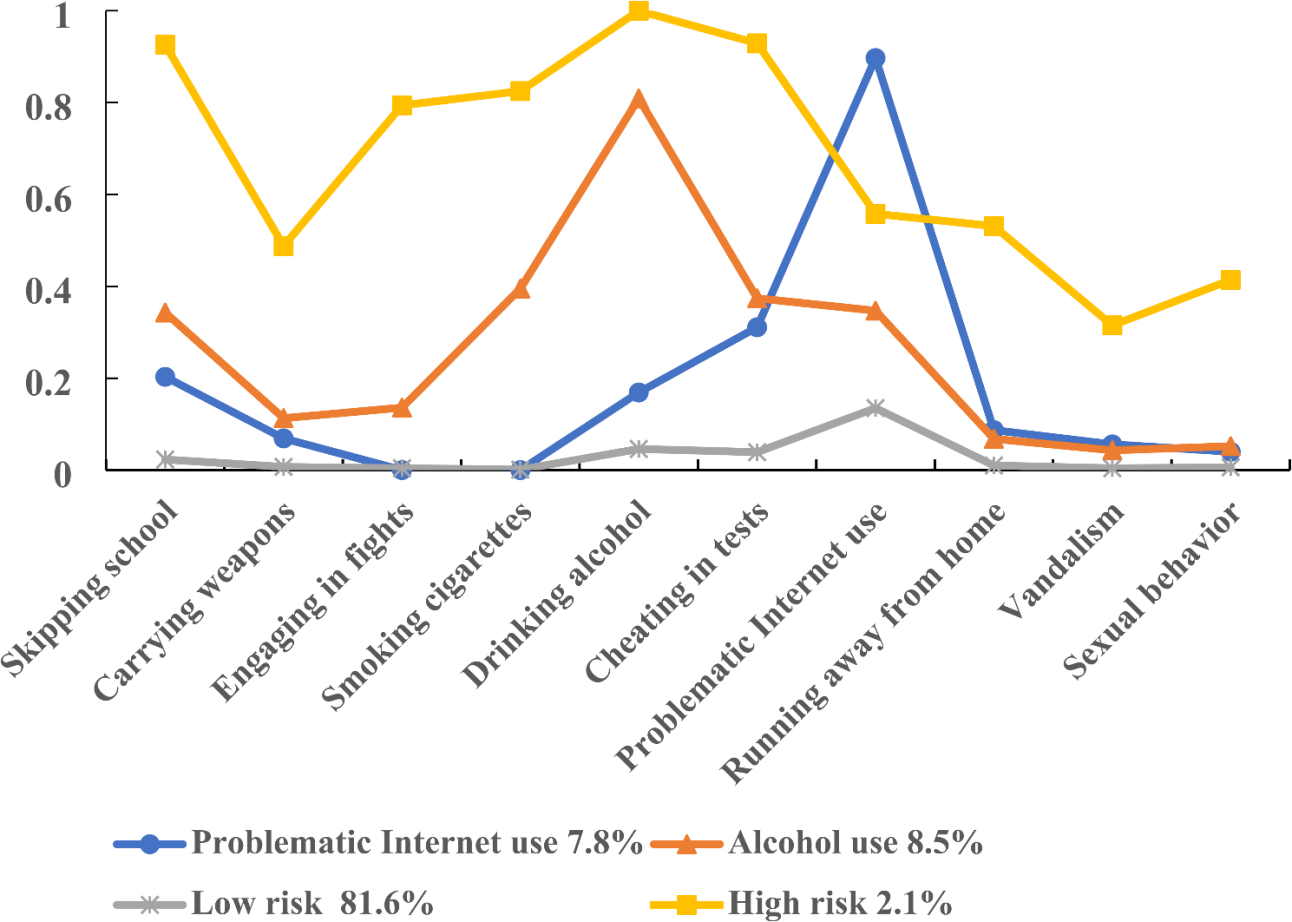
Patterns of health risk behaviors among Chinese adolescents

Four latent classes were identified: “Low risk” (81.6%), “Problematic Internet use” (7.8%), “Alcohol use” (8.5%), and “High risk” (2.1%). The “Low risk” class was the largest one, marked by low probabilities of all health-risk behaviors. Individuals in the “Problematic Internet use” class had the highest probability of problematic Internet use (89.7%), but relatively low probabilities of other health-risk behaviors. Adolescents in the “Alcohol use” class were marked by a high probability of drinking alcohol (80.9%), but relatively low endorsement of other health-risk behaviors. Members of the “High risk” subgroup reported relatively high probabilities of involvement in seven of ten health-risk behaviors (> 50%), and probabilities of carrying weapons, vandalism and sexual behavior were higher than other classes (Table 3; Fig. 1).

**Table 3:** Four-latent-class model of health-risk behavior (*n* = 1607)

Assigned label	Low risk	Problematic Internet use	Alcohol use	High risk
Latent class prevalence	1311 (81.6%)	126 (7.8%)	136 (8.5%)	34 (2.1%)
Item-response probabilities				
Skipping school	0.023	0.203	0.343	**0.926**
Carrying weapons	0.007	0.069	0.113	0.488
Engaging in fights	0.004	0.000	0.136	**0.794**
Smoking cigarettes	0.001	0.000	0.395	**0.825**
Drinking alcohol	0.046	0.169	**0.809**	**1.000**
Cheating in tests	0.039	0.311	0.374	**0.929**
Problematic Internet use	0.135	**0.897**	0.347	**0.558**
Running away from home	0.010	0.087	0.068	**0.531**
Vandalism	0.004	0.056	0.043	0.315
Sexual behavior	0.006	0.040	0.052	0.414

Note: Item-response probabilities > 0.5 in bold to facilitate interpretation

### The risk and protective factors of latent class membership

As displayed in Table 4, in the logistic regression, the “Low risk” class was designated as the reference group. Relative to the “Low risk”, adolescents with higher levels of sensation seeking, deviant peer affiliation, and childhood abuse were more likely to be assigned to the “Problematic Internet use” class, while those with high degrees of parental monitoring and school connectedness were less likely to be in the “Problematic Internet use” class. Likely, those with higher levels of sensation seeking and deviant peer affiliation, lower scores of parental monitoring and school connectedness were more likely to be assigned to the “Alcohol use” class, compared to the “Low risk”. Additionally, males were associated with increased odds of being in the “Alcohol use” and “High risk” classes, relative to the “Low risk”. Moreover, students in the “High risk” class were more prone to report higher levels of sensation seeking, deviant peer affiliation, and childhood abuse, but lower degrees of parental monitoring and school connectedness than the “Low risk” class.

**Table 4:** Adjusted odds ratios for factors predicting latent class membership (Reference = Low risk, *n* = 1599)

Variables	Problematic Internet use	Alcohol use	High risk
	aOR (95% CI)	aOR (95% CI)	aOR (95% CI)
***Individual level***			
Age	1.08 (0.82 ~ 1.42)	1.38 (1.05 ~ 1.81)^*^	1.88 (1.07 ~ 3.31)
Sex (ref: female)	0.74 (0.49 ~ 1.11)	2.50 (1.54 ~ 4.05)^***^	3.29 (1.06 ~ 10.29)^*^
Grade (ref: 11)	1.13 (0.71 ~ 1.79)	1.01 (0.63 ~ 1.62)	1.40 (0.53 ~ 3.68)
Living places (ref: rural)	1.18 (0.76 ~ 1.83)	1.55 (0.99 ~ 2.43)	1.66 (0.67 ~ 4.17)
Sensation seeking	1.09 (1.04 ~ 1.14)^***^	1.15 (1.09 ~ 1.20)^***^	1.25 (1.14 ~ 1.37)^***^
***Peer level***			
Deviant peer affiliation	1.15 (1.09 ~ 1.22)^***^	1.23 (1.17 ~ 1.30)^***^	1.41 (1.28 ~ 1.55)^***^
***Family level***			
Maternal education (Ref: College or above)			
Elementary school or below	0.67 (0.31 ~ 1.55)	0.50 (0.23 ~ 1.08)	0.46 (0.08 ~ 2.51)
Junior high school	0.98 (0.48 ~ 1.98)	0.62 (0.31 ~ 1.25)	1.41 (0.32 ~ 6.27)
Senior high school	0.80 (0.40 ~ 1.60)	0.57 (0.29 ~ 1.11)	1.96 (0.49 ~ 7.91)
Intact family (Ref: Yes)	1.95 (1.01 ~ 3.77)^*^	0.97 (0.47 ~ 2.04)	1.09 (0.31 ~ 3.86)
Paternal education (Ref: College or above)			
Elementary school or below	1.00 (0.41 ~ 2.43)	2.00 (0.87 ~ 4.57)	0.88 (0.14 ~ 5.52)
Junior high school	1.06 (0.53 ~ 2.10)	1.17 (0.60 ~ 2.31)	1.41 (0.38 ~ 5.20)
Senior high school	0.91 (0.47 ~ 1.76)	0.95 (0.50 ~ 1.83)	1.13 (0.31 ~ 4.05)
Intact family (Ref: No)	0.53 (0.27 ~ 1.02)	0.86 (0.41 ~ 1.80)	0.84 (0.24 ~ 2.92)
Household dysfunction (Ref: No)	0.89 (0.56 ~ 1.40)	1.15 (0.74 ~ 1.79)	1.68 (0.67 ~ 4.20)
Parental monitoring	0.96 (0.92 ~ 0.99)^*^	0.92 (0.89 ~ 0.95)^**^	0.90 (0.84 ~ 0.95)^**^
Childhood abuse	1.11 (1.05 ~ 1.16)^***^	1.05 (0.99 ~ 1.11)	1.13 (1.05 ~ 1.20)^**^
***School level***			
School connectedness	0.95 (0.92 ~ 0.98)^**^	0.95 (0.93 ~ 0.98)^**^	0.94 (0.89 ~ 0.99)^*^

Note: aOR, Adjusted Odds Ratios; CI, Confidence Interval. ^***^p < 0.001, ^**^p < 0.01, ^*^p < 0.05

## Discussion

This study explored patterns of health-risk behaviors and contributed to the existing literature by analyzing the multi-level determinants of these patterns among Chinese adolescents through LCA. The current research identified four distinct subgroups: Low Risk (81.6%), Problematic Internet use (7.8%), Alcohol Use (8.5%), High Risk (2.1%) and found that individual, peer, family, and school factors significantly predicted latent patterns. This survey offers a valuable foundation for understanding the types and influencing factors of adolescents’ multiple risk behaviors, serving as a practical basis for future research and intervention development.

Congruent with previous research [51, 52], the findings suggested that adolescent health-risk behaviors were multiple and co-occurring. Prior surveys typically found three or four latent classes, commonly one class with relatively low odds of health-risk behaviors, and another one with high endorsement of all health-risk behaviors [14, 53]. Compared to previous literature, the current study identified a novel class (Problematic Internet use) marked by a high likelihood of problematic Internet use. Due to low self-control or self-discipline of adolescents, they may be particularly vulnerable to problematic Internet use in times of COVID-19 [54]. The lockdown measures and consequent lack of social interaction may increase the opportunities for prolonged and intensified using of the Internet, and the surge in adolescent problematic Internet use is a growing concern [55]. Problematic Internet use was the most prominent health-risk behavior in this study (23.9%), indicating that developing effective prevention of problematic Internet use is urgent. Therefore, moderate usage of the Internet should be promoted for adolescents. Also, parents need to improve the communication and monitoring of their child’s Internet behaviors.

Moreover, a vast number of factors can predict patterns of health-risk behaviors among Chinese adolescents at an individual, peer, family, and school level, which supports the view of the ecological framework that risk and protective factors interact at the micro, mezzo, and macro levels [16]. At the individual level, boys are more likely to belong to “Alcohol use” and “High risk” compared with girls, which is consistent with previous studies [1]. In line with traditional Chinese social and gender roles, risk-taking among females is viewed less favorably than among males. Besides, high-sensation-seeking adolescents are more likely to belong to the “Problematic Internet use”, “Alcohol use” and “High risk” groups. The positive association between sensation seeking and health-risk behaviors in Chinese context has been well documented [13]. Some scholars hold the view that the occurrence of risk behavior in adolescence was related to sensation seeking, convention challenging, and maturity demonstration, which will be manifested in higher rates of impulsive and health-risk behaviors [12, 18].

Consistent with empirical studies [56, 57], the finding suggested that deviant peer affiliation was associated with an increased adolescents’ engagement in health-risk behaviors. Adolescents spend increasing amounts of time with peers, and adolescence is a developmental period characterized by the desire to behave in ways of their friends [58]. Cambron and colleagues found that poor peer interactions independently predicted smoking and drinking behaviors in early adolescence [59]. Also, the Group Socialization Theory emphasized the external environment, especially peer groups, played a key role in environmental adaptation and behavior formation for children [60]. Understanding how deviant peer affiliation influence adolescent behaviors has vital implications for reducing their behavioral problems.

In terms of family level, the results revealed that childhood abuse and parental monitoring acted as significant predictors of adolescents’ behaviors. As noted by a study in 34 Quebec high schools, sexual abuse significantly predicted health-risk behaviors, including alcohol abuse, cannabis abuse, and delinquency [61]. Childhood abuse is related to widespread abnormalities in brain structure and function, which further leads to an increased susceptibility to a variety of health-risk behaviors [62]. Parental substance abuse, parental separation/divorce (indicators of household dysfunction) were associated with increased odds of smoking in U.S. adults after controlling for important confounders [63]. Parental monitoring was a vital contributor to patterns of adolescents’ multiple risk behaviors in The Bahamas [1]. Parental knowledge of students’ whereabouts, companions, and activities can prevent opportunities for involvement in risk behaviors or spending time with peers who might promote such behaviors [64]. These findings highlight the significance of reducing childhood abuse and fostering parental monitoring to support healthy adolescent development.

Adolescents in the problematic Internet use, alcohol use and high risk classes reported low levels of school connectedness than those in the low risk class. A survey documented that school connectedness reduced involvement in problematic Internet use among Chinese students [65]. Data from the 2021 nationally representative Youth Risk Behavior Survey showed that school connectedness was negatively related to risk behaviors among U.S. high school students [66]. A longitudinal study also found the protective effects of school connectedness on alcohol use among adolescents [67]. This study emphasize school connectedness will protect against multiple adolescent health risks, and school-based strategies should promote safe and supportive environments for students.

During the time of our data collection, the COVID-19 pandemic caused widespread disruptions to school operations and increased stress and trauma for some adolescents. These results have crucial implications for prevention and intervention of adolescent risk behaviors in the context of a pandemic and increased adversity. First, fostering collaboration among individuals, peers, families, and schools is essential for reducing adolescent risk behaviors. Comprehensive, multi-component interventions are likely to be more effective than single-component interventions. Second, targeted interventions and professional support should be prioritized for vulnerable adolescents, particularly those exposed to factors such as childhood abuse, etc.

### Limitations

The following limitations should be noted in this study. First, all adolescents were from a single city in China and the sample size was relatively small, which may limit the generalizability of these findings. Second, as the questionnaire included sensitive topics, peer, family, and school variables were assessed from the adolescents’ perspectives. This approach may introduce social desirability bias, leading to potential underestimate of risk behaviors. Third, the cross-sectional design of this survey did not allow for causal inferences among the variables. Fourth, this study was conducted in only four senior high schools focusing on grades 10 and 11, which may introduce selection bias. Fifth, this study was conducted in 2021 during the COVID-19 pandemic, thus, the results and implications may differ from findings obtained before and after the pandemic. Nevertheless, these findings, despite being based on older data, provide valuable insights for developing prevention and intervention strategies for adolescents in the context of a pandemic and increased adversity. Therefore, to untangle these limitations, future studies should employ longitudinal designs using nationally representative samples, and data from parents, peers, and teachers should be added.

## Conclusions

This study identified patterns of multiple health-risk behaviors among Chinese high school students and found that multi-level individual and social factors affected latent classes of adolescent health-risk behaviors. These findings may provide clues for designing effective interventions to reduce health-risk behaviors among adolescents.

## Data Availability

Data are available from the corresponding author.
